# Arabidopsis microRNA expression regulation in a wide range of abiotic stress responses

**DOI:** 10.3389/fpls.2015.00410

**Published:** 2015-06-04

**Authors:** Maria Barciszewska-Pacak, Kaja Milanowska, Katarzyna Knop, Dawid Bielewicz, Przemyslaw Nuc, Patrycja Plewka, Andrzej M. Pacak, Franck Vazquez, Wojciech Karlowski, Artur Jarmolowski, Zofia Szweykowska-Kulinska

**Affiliations:** ^1^Department of Gene Expression, Faculty of Biology, Institute of Molecular Biology and Biotechnology, Adam Mickiewicz UniversityPoznan, Poland; ^2^Zurich-Basel Plant Science Center, Swiss Plant Science Web, Botanical Institute, University of BaselBasel, Switzerland; ^3^Department of Computational Biology, Faculty of Biology, Institute of Molecular Biology and Biotechnology, Adam Mickiewicz UniversityPoznan, Poland

**Keywords:** miRNA, pri-miRNA, abiotic stress, gene expression

## Abstract

Arabidopsis microRNA expression regulation was studied in a wide array of abiotic stresses such as drought, heat, salinity, copper excess/deficiency, cadmium excess, and sulfur deficiency. A home-built RT-qPCR mirEX platform for the amplification of 289 Arabidopsis microRNA transcripts was used to study their response to abiotic stresses. Small RNA sequencing, Northern hybridization, and TaqMan® microRNA assays were performed to study the abundance of mature microRNAs. A broad response on the level of primary miRNAs (pri-miRNAs) was observed. However, stress response at the level of mature microRNAs was rather confined. The data presented show that in most instances, the level of a particular mature miRNA could not be predicted based on the level of its pri-miRNA. This points to an essential role of posttranscriptional regulation of microRNA expression. New Arabidopsis microRNAs responsive to abiotic stresses were discovered. Four microRNAs: miR319a/b, miR319b.2, and miR400 have been found to be responsive to several abiotic stresses and thus can be regarded as general stress-responsive microRNA species.

## Introduction

Plants are constantly challenged by a complex array of environmental stresses that require their fast and proper response in order to adapt and survive. The main abiotic stresses that affect plants and crops in the field include drought, salinity, heat, cold, chilling, freezing, nutrient, high light intensity, ozone (O_3_), and anaerobic stresses (Suzuki et al., 2014). Morphological and physiological adaptations to abiotic stresses require complex rearrangements of gene expression networks controlled at the transcriptional and posttranscriptional level. Recent studies have shown a crucial regulatory role of microRNAs in plant response to environmental cues. Many different microRNAs have been found to be responsive to various abiotic stresses in different plant species of economic importance like rice, barley, wheat, sugarcane, legumes, tomato, potato and in many other species (Kruszka et al., 2012, 2014; Carnavale Bottino et al., 2013; Pieczynski et al., 2013; Cao et al., 2014; Naya et al., 2014; Pandey et al., 2014; Guerra et al., 2015; Nigam et al., 2015; Zhang, 2015). MicroRNAs act at the posttranscriptional level and guide target mRNA cleavage or translation inhibition (Bartel, 2004; Brodersen et al., 2008; Beauclair et al., 2010). Thus, they are general negative regulators of gene expression. The microRNA genes are transcribed by RNA polymerase II (Pol II) (Rogers and Chen, 2013). Bioinformatic tools identified abiotic response elements in the promoter region of many microRNA genes. Abiotic response elements were found using bioinformatic tools in the promoter region of many microRNA genes. This suggests transcriptional regulation of at least some microRNA genes (Higo et al., 1999; Megraw et al., 2006; Zhao et al., 2013). Primary miRNA transcripts (pri-miRNAs) form a characteristic stem and loop structure in which microRNA and its cognate so called, microRNA star (^*^) are embedded. The microRNA and microRNA^*^ duplex is excised from the hairpin by the action of a microprocessor complex in which DICER-LIKE 1 protein (DCL1) plays the most important role. The majority of plant pri-miRNAs represents independent transcriptional units harboring multiple introns (Rogers and Chen, 2013; Szweykowska-Kulińska et al., 2013). These transcripts undergo extensive posttranscriptional maturation processes like splicing, alternative splicing, and alternative polyadenylation site selection. Currently, the reports showing the importance of the posttranscriptional regulation of microRNA biogenesis are indeed accumulating (Bielewicz et al., 2012; Yan et al., 2012; Jia and Rock, 2013). The efficiency of the conversion of a pri-miRNA to a pre-miRNA stem-loop structure and of further steps that lead to the production of mature microRNAs can also be regulated and dependent on the level of proteins involved in microRNA biogenesis (Vazquez et al., 2004; Kurihara et al., 2006; Dong et al., 2008; Laubinger et al., 2008; Manavella et al., 2012; Ren et al., 2012; Zhan et al., 2012; Kruszka et al., 2013; Rogers and Chen, 2013; Wang et al., 2013). Altogether these data suggest that microRNA level can be regulated transcriptionally as well as posttranscriptionally. The aim of this study was to reveal if a correlation exists between the levels of pri-miRNAs and miRNAs upon stress application. In this paper we show that the level of a particular pri-miRNA is not predictive for the level of its cognate microRNA.

The number of newly discovered Arabidopsis miRNAs is continually growing. For example, miRBase release 18 contained 291 known microRNAs for *A. thaliana* while the miRBase release 21 contained 427 microRNAs (Griffiths-Jones, 2004; Kozomara and Griffiths-Jones, 2014). Many of these recently discovered new microRNAs were not tested for their responsiveness to environmental changes. Thus, the relationship between Arabidopsis miRNA expression and stress response is still an attractive area to be explored. As a result of this study, multiple new stress-responsive microRNAs were identified.

The performed studies also enabled the identification of Arabidopsis multi-stress responsive microRNAs. We found miR319a/b, miR319b.2, and miR400 as multiple stress-responsive microRNAs. This would suggest a complex regulatory network involved in controlling the level of the same microRNA under different abiotic stresses.

## Materials and methods

### Plant material and growth conditions

*Arabidopsis thaliana* (L.) Heynh, Col-0 wild type plants and homozygous line Δ*miR319b* (SALK_037093) (Sobkowiak et al., 2012) were used in the experiments. The wild type plants were grown after stratification at 16 h/8 h day/night cycles at 22°C on 0.8% agar ½MS plates for the following abiotic stress conditions: 250 mM NaCl excess, 10 μM Cu excess, 10 μM Cd excess, Cu deficiency, and S deficiency (Figure S1A, upper panel). The stresses were applied for 14-day old seedlings in ½MS liquid medium for 24 h. For heat stress, different sets of plates containing 14-day old seedlings were transferred into 37°C condition for 0.5 and 6 h. For salinity, Cu excess and Cd excess stress inductions up to 250 mM NaCl, 10 μM CuSO_4_ and 10 μM CdSO_4_ were added to ½MS liquid media, respectively (Figure S1A, upper panel). For Cu and S deficiency stresses, 0.1 μM CuSO_4_^*^5H_2_O was eliminated and all sulfates were replaced by equivalent chloride salts, respectively. For the progressive drought experiment, after stratification *Arabidopsis thaliana* (L.) Heynh, Col-0 wild type plants were grown at 16 h, 22°C/8 h, 15°C day/night cycles in 70% SWC (soil water content) soil in a MTPS 144 (Conviron, Winnipeg, Canada) controlled environment at 50–60% relative humidity and 200 μmol· m^−2^·s^−1^ light intensity (Figure S1A, lower panel). The stress was applied at 1.10 growth stage (Boyes et al., 2001) by water withholding, and continued until the soil moisture level reached 30% SWC (3 days before wilting) and 20% SWC (wilting), both for three different plant batches. The experiment was monitored by the leaf relative water content (RWC) measurements (Weatherly, 1951). All stress experiments were done in three biological replicates.

### RNA isolation

For quantitative real-time PCR analyses, including TaqMan® miRNA assays (ABI, Life Technologies, USA), total RNA was isolated from 100 mg of 15-day old seedlings and 1.13 growth stage (Boyes et al., 2001) plant leaves using TRIzol Reagent (Invitrogen, Karlsruhe, Germany) and Direct-zol™ RNA MiniPrep Kit (ZymoResearch Corp., USA). RNA concentration measurements, RNA integrity determination, and DNA contamination removal were done as previously described (Szarzynska et al., 2009). For Northern blot analyses, 20 μg of total RNA and 60 μg of total RNA were used for the detection of ath-miRNA319 and b.2, respectively. Total RNA was isolated from 100 mg of 15-day old seedlings and 1.13 growth stage (Boyes et al., 2001) plant leaves using TRIzol Reagent (Invitrogen, Karlsruhe, Germany) and subsequent double chloroform extractions and isopropanol precipitation as previously described (Kruszka et al., 2013). For small RNA NGS analyses, total RNA enriched with small RNA was isolated from 100 mg of 15-day old seedlings and 1.13 growth stage (Boyes et al., 2001) plant leaves using a protocol as previously described (Kruszka et al., 2013). NGS dedicated RNA was verified with regard to its concentration and integrity using Agilent RNA 6000 Nano Kit (Agilent Technologies, Inc., Waldbronn, Germany).

### Northern blot analyses

RNA electrophoresis, blot transfer, and hybridization were performed as previously reported (Kruszka et al., 2013). All hybridizations were performed in three biological repetitions. DNA oligo probes [γ-^32^P] ATP labeling, loading control usage, blots exposition, scanning, and quantification were done as previously described (Kruszka et al., 2014). Oligonucleotide DNA probes, listed in Supplementary data Table S1, were complementary to particular *Arabidopsis thaliana* mature miRNAs. A probe complementary to U6 snRNA was used as a loading control (Szarzynska et al., 2009).

### Quantitative real-time PCR profiling of pri-miRNAs, miRNAs, and stress marker mRNAs

Three microgram of DNA-free RNA was used for real-time PCR analysis carried out as described in (Kruszka et al., 2013). All measurements were carried out in three biological replicates. The efficiency of cDNA synthesis was assessed by qPCR amplification of GAPDH (At1g13440) 5′ and 3′ cDNA fragments. For qPCR analysis, cDNA was diluted four times to total volume of 1 μl. Ct values for all miRNA primary transcripts and other transcript cDNAs were normalized to the GAPDH3′cDNA fragment Ct value. The stress application was confirmed by the amplification of a given stress marker mRNAs. Figure S1B shows the induction or downregulation of the marker mRNAs. For TaqMan® miRNA assays (ABI, Life Technologies, USA), 10 ng of DNA-free RNA was used for each reverse transcription reaction performed using TaqMan® MicroRNA Reverse Transcription Kit (ABI, Life Technologies, USA) according to the manufacturer's protocol. The TaqMan® miRNA assays were performed using TaqMan® Universal PCR Master Mix II, with UNG (ABI, Life Technologies, USA) according to the manufacturer's protocol. Figure S2 shows the increased or decreased levels of particular miRNAs measured using TaqMan® miRNA assays (ABI, Life Technologies, USA) and the expression up- or downregulations of the respective pri-miRNAs determined by modified mirEX qPCR platform (Bielewicz et al., 2012, http://comgen.pl/mirex2/). The primers for pri-miRNAs qPCR can be found at www.comgen.pl/mirex2/ website. The list of qPCR primers for stress marker genes and TaqMan® miRNA assay probes can be found in the Supplementary data Table S1.

### 5′RACE experiments

5′RACE experiments were performed using SMARTer RACE cDNA Amplification Kit (Clontech, Mountain View, CA, USA) according to the manufacturer's protocol. All primers used in the experiments are listed in Table S1 (Supplementary data). PCR reactions, cloning and sequencing of 5′RACE products were done as previously reported in (Kruszka et al., 2013).

### Small RNA sequencing and bioinformatic analyses

*Arabidopsis thaliana* total RNA enriched with small RNA and isolated from stress conditioned plants was used for small RNA library generation and sequencing by Illumina HiSeq 2000 system (Illumina Inc., San Diego, CA, USA), performed by BGI Tech Solutions, Hongkong, Co., Limited. All small RNA sequencing reactions were carried out in three biological replicates. Table 1 shows read numbers obtained for small RNA sequencing samples used in our studies.

**Table 1:** **Read numbers summary for small RNA sequencing of stress samples**.

**Sample name**	**Arabidopsis Col-0 growth stage**	**Stress samples (each in triplicate)**	**Sum of number of reads for triplicate**	**Sum of number of reads mapped to genome for triplicate**	**% of reads mapped to genome**	**Sum of number of reads mapped to mirBase for triplicate**	**Average number of identified miRNAs in each triplicate**
C30	27 days old adult plants	Control for 30%SWC drought	31523627	29683421	94.16245	10521274	310
D30	27 days old adult plants	30%SWC drought	32029415	31098843	97.09463	9615205	315
C20	30 days old adult plants	Control for 20%SWC drought	32043396	30485933	95.13952	9590740	305
D20	30 days old adult plants	20%SWC drought	31825087	30994515	97.3902	8669427	308
H 0	15 days old seedlings	Control for heat stresses	31860029	30697515	96.35118	14056686	322
H 0.5	15 days old seedlings	0.5 h heat (37°C)	31703864	30999560	97.77849	14624263	313
H 6	15 days old seedlings	6 h heat (37°C)	31422415	30277919	96.35771	13663612	307
C	15 days old seedlings	Control for NaCl excess, Cu deficiency, Cu excess, Cd excess, sulfur deficiency stresses	31974880	31552714	98.67969	12455085	319
NaCl+	15 days old seedlings	24 h NaCl [250 mM] excess	31202256	30834070	98.82	4115278	299
Cu−	15 days old seedlings	24 h copper deficiency	31652107	31314891	98.93462	13426107	320
Cu+	15 days old seedlings	24 h copper [10 μM] excess	31453075	31075237	98.79872	13059369	325
Cd+	15 days old seedlings	24 h cadmium [10 μM] excess	31176242	30668394	98.37104	11940906	311
S−	15 days old seedlings	24 h sulfur deficiency	31662380	31250642	98.6996	12717241	322

Each sample was mapped first to *A. thaliana* genome using Bowtie program to check if obtained reads were derived from the plant studied. The mapping quality was 97–98% for each sample (Langmead et al., 2009). Afterwards the reads were mapped to mature miRNA sequences obtained from miRBase (release 21) as described below. For each sample and each replicate roughly 300 miRNAs (from 290 for 3rd replicate in NaCl excess stress to 330 in 2nd replicate from 24 h sulfur deficiency stress) were identified.

The reads obtained from the small RNA next generation sequencing (NGS) were mapped to all mature *Arabidopsis thaliana* miRNAs found in miRBase (release 21) (Kozomara and Griffiths-Jones, 2014) additionally supplemented with four sequences not deposited in miRBase (miR319a.2, miR319b.2, miR319c.2, and miR163.2) (Sobkowiak et al., 2012; Jeong et al., 2013) using script countreads_mirna.pl from the analysis package described herein (Eminaga et al., 2013). The Perl script first eliminates the sequences shorter than 17 nucleotides and then aligns the sequences to reference mature miRNA sequences. To maximize read counts and to incorporate reads representing putative isomiRs, an unmatched, full length sequence is trimmed at both ends before another matching attempt (Eminaga et al., 2013). The script was applied to each fastq file for every biological replicate from every condition. Afterwards another script from the same analysis package (cmp_mirna.pl) was used to normalize read counts by dividing the total counts for each particular miRNA by the total counts of the sample and multiplying by 10^6^ (counts per million). The triplicate measurements were averaged. Subsequently, an in house—generated python script was used to calculate fold change and *p*-value between every condition and its control. *P*-value was calculated with f_oneway function from Python scipy.stats (version 0.15.1) module that performs a One-Way Anova test using data for every replicate from the given condition and every replicate from the control (to include standard deviation). The described method has been chosen instead of using standard analysis with the usage of R-package DESeq (Anders and Huber (2010) as this method gave more reliable results confirmed by Northern hybridization than the procedure based on R. The data used in this study have been deposited under NCBI GEO accession GSE66599. Target prediction was previously performed in (Sobkowiak et al., 2012).

## Results

### Abiotic stress regulation of *Arabidopsis thaliana* microtranscriptome

**Figure 1:**
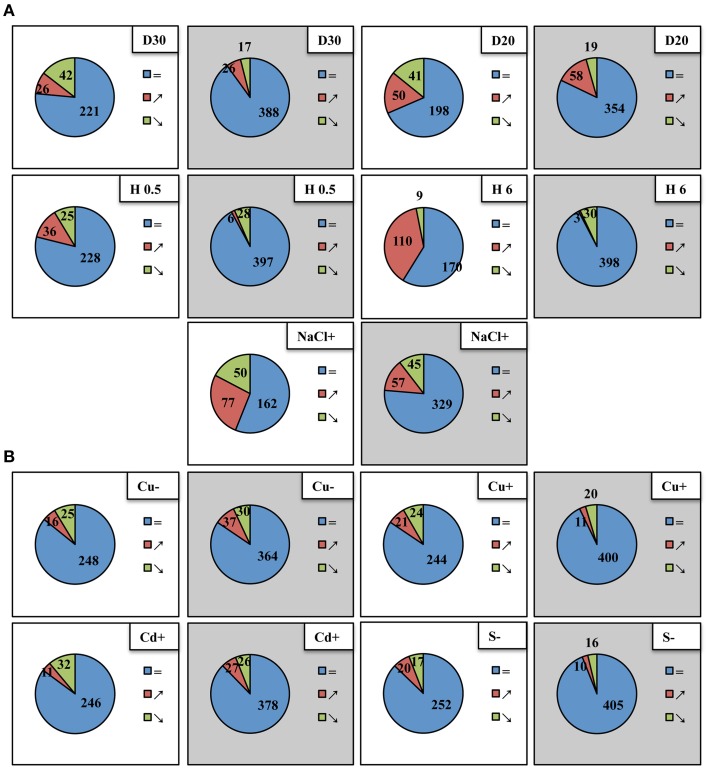
**Differential expression of** ***Arabidopsis thaliana*** **pri-miRNAs (white background) and mature miRNAs (gray background) under different abiotic stress conditions**. Blue, red, and green colors indicate pri-miRNAs and mature miRNAs unchanged, upregulated and downregulated, respectively. **(A)** Upper panel depicts 30%SWC and 20%SWC drought stress (D30 and D20, respectively), middle panel shows half an hour and 6 h heat stress (H 0.5; H 6), lower panel shows 250 mM salinity (NaCl+), **(B)** upper panel presents copper deficiency (Cu−) and 10 μM copper excess (Cu+), lower panel depicts 10 μM cadmium excess (Cd+) and sulfur deficiency (S−). The fold change-based numbers of up- and downregulated pri-miRNAs and mature miRNAs shown in Venn diagrams were suggested by a two-tailed Student *t* test (*p* ≤ 0.05) for pri-miRNA RT-qPCR analyses (modified mirEX high throughput real-time PCR platform, Bielewicz et al., 2012, http://comgen.pl/mirex2/) and a One-Way Anova test (*p* ≤ 0.05) for mature miRNA analyses by small RNA NGS, respectively.

**Figure 2:**
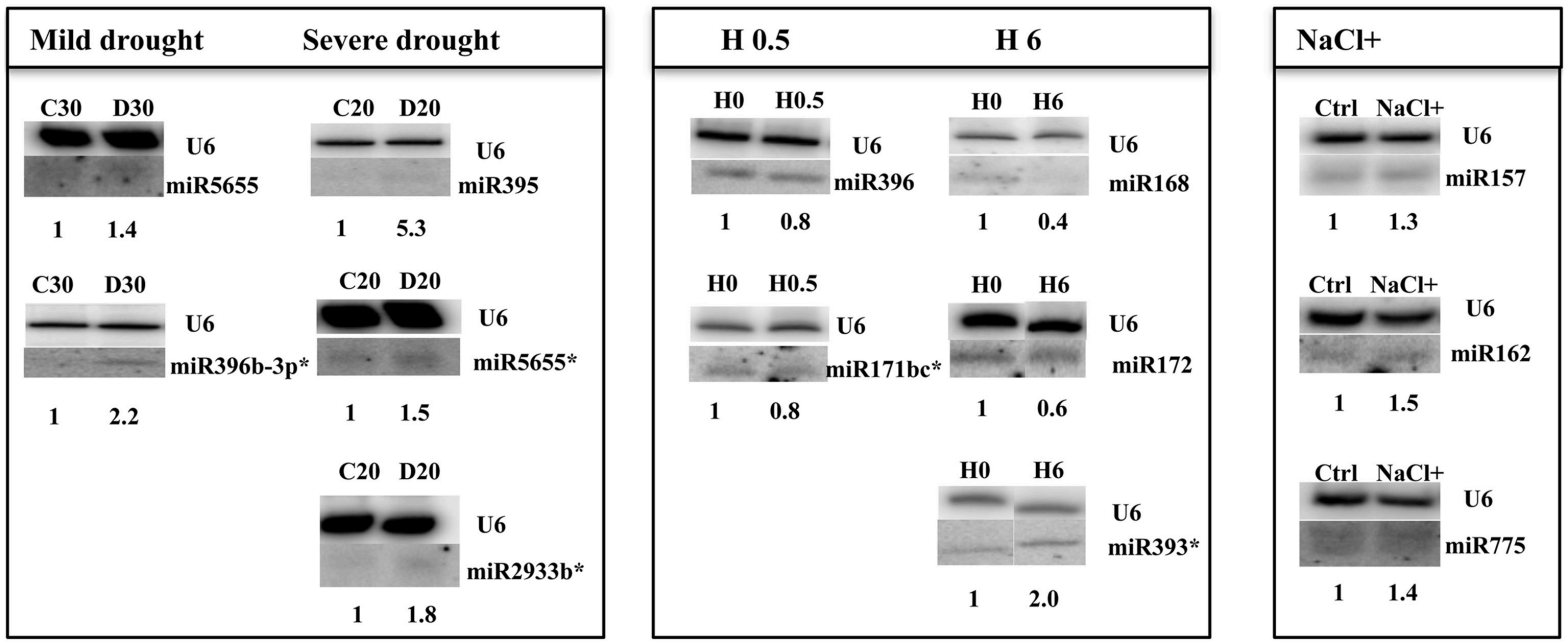
***Arabidopsis thaliana*** **miRNAs revealed by Northern hybridization as affected under 30%SWC mild drought, 20%SWC severe drought, 0.5 h heat, 6 h heat, and salinity stress conditions**. Only new stress responsive miRNAs unknown until now are shown. The white lines separate signals from miRNAs and U6snRNA loading control probed on the same blots. H 0 and H 6 samples were run on the same gel but not next to each other as indicated by the white separating lines between all signals probed. The star marks NGS revealed miRNAs with no statistic significance and the maintained tendency of expression level change as seen in Northern hybridization. Symbols representing various stresses are marked as described in the Figure 1.

**Figure 3:**
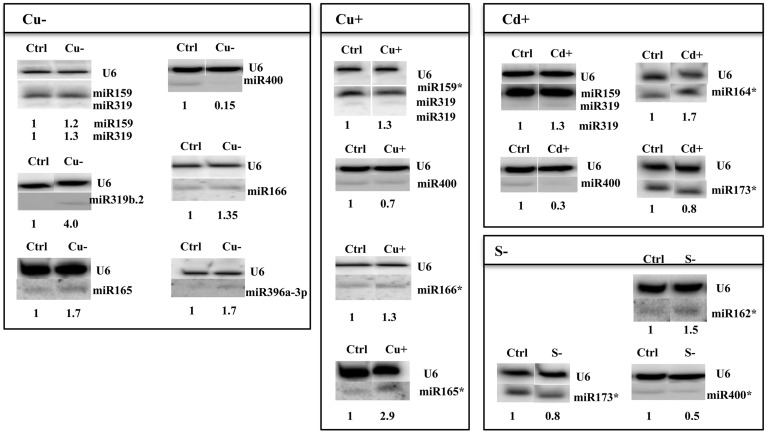
***Arabidopsis thaliana*** **miRNAs revealed by Northern hybridization as affected under copper deficiency, copper excess, cadmium excess, and sulfur deficiency stress conditions**. Only new stress responsive miRNAs unknown until now are shown. The white lines separate signals from miRNAs and U6snRNA loading control probed on the same blots. Control (Ctrl) and stress (Cu−, Cu+, Cd+, S−) samples were run on the same gel but not next to each other as indicated by the white separating lines between all signals probed. The star marks NGS revealed miRNAs with no statistic significance and the maintained tendency of expression level change as seen in Northern hybridization. Symbols representing various stresses are marked as described in the Figure 1.

A high throughput real-time PCR platform, mirEX (modified mirEX platform, Bielewicz et al., 2012, http://comgen.pl/mirex2/) was applied to discriminate between 289 individually known primary miRNA precursors (pri-miRNAs) and to reliably analyze their individual expression patterns under different abiotic stress conditions. Table S2 presents the RT-qPCR data of all pri-miRNAs studied. Total RNA isolated from plants subjected to various abiotic stresses was also analyzed with regard to mature miRNA level utilizing small RNA NGS approach (Figure 1). The abundance of the selected microRNAs was tested using Northern hybridization (for detailed data see Tables S2, S3 and Figures 2, 3) as well as TaqMan® miRNA assays (ABI, Life Technologies, USA) (Figure S2).

#### *Arabidopsis thaliana* pri-miRNAs and mature miRNAs expression under different abiotic stress conditions

From the array of abiotic stresses studied, a broad transcriptional response of pri-miRNAs was clearly visible for common climate change related stresses, like drought, heat, and salinity (Figure 1A). Approximately, the same number of downregulated pri-miRNAs as well as mature miRNAs was observed under mild and severe drought. However, under severe drought conditions, the number of upregulated pri-miRNAs and mature miRNAs doubled in comparison to the mild drought conditions. For heat stress experiments the most profound upregulation of pri-miRNAs was observed after 6 h of heat duration. Surprisingly, the number of mature microRNAs that were up- or downregulated in two time points of heat stress was rather low (Figure 1A). Under salinity stress condition, expression changes were found in nearly half of the pri-miRNAs analyzed while only one fourth of the mature microRNAs were affected (Figure 1A). The transcriptional response of pri-miRNAs to the copper, cadmium and sulfur stresses applied was quite limited compared to the stresses mentioned above (Figure 1B). The number of mature miRNAs responding to sulfur deficiency and copper excess was lower than the number of pri-miRNAs responsive to these stresses. On the contrary, upon copper deficiency and cadmium excess the number of responsive mature miRNAs was higher than the number of responsive pri-miRNAs (Figure 1B). These results suggest that there is no direct correlation between the level of pri-miRNAs and mature miRNAs during plant response to abiotic stresses.

#### Individual pri-miRNA—miRNA relationships under different abiotic stresses

For the drought stress affected pri-miRNAs, their down- and upregulation was often connected with unchanged levels in their cognate miRNAs (Table 2). However, for the pri-miRNA transcripts expressed at a constant level under drought stress, the increased levels of their cognate miRNAs were mostly detected.

**Table 2:** **Individual pri-miRNA—miRNA relationships under different abiotic stresses**.

**Pri-miRNA**—**miRNA relationships**		**D30**	**D20**	**H 0.5**	**H 6**	**NaCl+**	**Cu−**	**Cu+**	**Cd+**	**S−**
Pri-miRNA > miR	Pri-miRNA 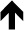 , miR const.	32	47	46	141	80	16	23	10	26
	Pri-miRNA 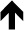 , miR 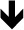	2	−	2	(^*^) 22	7	1	−	1	−
	Pri-miRNA const., miR 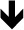	14	14	17	4	25	26	18	20	14
Pri-miRNA < miR	Pri-miRNA const., miR 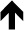	18	29	4	1	29	31	9	24	5
	Pri-miRNA 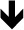 , miR 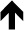	5	4	−	1	13	3	−	2	−
	Pri-miRNA 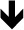 , miR const.	46	57	31	6	49	25	23	32	16
Pri-miRNA = miR	Pri-miRNA 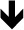 , miR 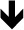	7	5	7	1	10	3	1	2	1
	Pri-miRNA 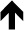 , miR 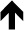	1	22	2	1	15	1	2	−	2

The p-value ≤ 0.05 was considered for the expression changes analyzed. The star depicts high number of expression changes observed for so called miRNA^*^ small RNAs.

Under heat stress conditions, the majority of pri-miRNAs were upregulated while their cognate miRNAs remained unchanged. Surprisingly, if any changes in mature miRNA abundance were detected, they referred mostly to so-called microRNA stars that mainly decreased after 6 h of heat stress. The pri-miRNAs, for the star small RNAs mentioned, were usually highly transcribed (Table S2).

More pri-miRNA transcripts were upregulated in response to salinity stress compared to drought stress but less when compared with 6 h of heat stress. Their cognate miRNAs remained mostly constant. The same relationship was true in the case of pri-miRNAs with downregulated level upon salinity stress (Table 2).

In instances involving metal and sulfur stress, similar relationships between the levels of pri-miRNAs and their cognate miRNAs were observed (Table 2). Altered levels of miRNAs were often accompanied by stable levels of their transcripts. Additionally, altered levels of pri-miRNAs were often accompanied by stable levels of their cognate miRNAs (Table 2).

The results show that it is difficult to predict the amount of a given microRNA relying only on its pri-miRNA expression pattern. This observation was supported by additional analyses of 10 randomly selected mature miRNAs, which levels were measured using TaqMan® miRNA assays (ABI, Life Technologies, USA) (Figure S2) (RT-qPCR). The obtained microRNA levels were compared to the level of cognate pri-miRNAs determined using RT-qPCR approach. The comparison of the level of pri-miRNAs and their cognate mature miRNAs showed (i) opposite changes in the levels of pri- and mature miRNAs, (ii) increased and decreased levels of miRNAs for constant pri-miRNA levels (iii) as well as increased and decreased levels of the pri-miRNAs for stable levels of mature miRNAs (Figure S2). These discrepancies can be due to extensive posttranscriptional regulation of the mature microRNA levels or due to different stabilities of various microRNAs. However, it has to be mentioned that we also found examples for a correlation between the level of pri-miRNA and its miRNA (see Table 2).

### Abiotic stress responsive novel Arabidopsis microRNAs

#### Drought-responsive new microRNAs

Using the NGS Illumina approach, it was determined that the mature miRNA response to severe drought conditions is generally broader compared to mild drought conditions (Tables S3A,B). Table 3 presents microRNAs with the most profoundly changed expression patterns that were determined using NGS and computed by One-Way Anova test (*p* ≤ 0.05). The number of microRNAs affected only under severe drought is more than doubled compared to microRNAs changed only under mild drought conditions. Among expression profile changes, the increase of a given miRNA level is predominant (Table 3).

**Table 3:** **30%SWC (D30) and 20%SWC (D20) drought stress affected** ***Arabidopsis*** **miRNAs revealed by high-throughput small RNA NGS**.

**D30**		**D20**			
**MiRNA**	**FC**	**MiRNA**	**FC**	**MiRNA**	**FC**
miR156h	1.40	miR156a-3p	1.82	miR408-3p	4.08
miR169e,d,f-5p,g-5p	1.72	miR156h	1.71	miR447c-5p	4.04
miR398b-5p,c-5p	3.08	miR159a	2.60	**miR773a**	1.96
miR399b,c-3p	1.61	**miR160a-5p,b,c-5p**	3.34	**miR823**	1.85
miR408-5p	1.83	**miR160a-3p**	0.35	**miR824-3p**	2.19
miR408-3p	1.65	**miR164c-5p**	1.83	**miR827**	1.73
**miR447a.2-3p**	2.45	miR169d,e,f-5p,g-5p	2.24	**miR848**	1.55
**miR846-5p**	2.21	miR171c-5p	0.49	**miR851-3p**	0.38
**miR846-3p**	1.63	**miR319a.2**	0.47	**miR852**	1.52
**miR854a,b,c,d,e**	0.49	**miR319b.2**	0.60	**miR858a**	2.89
**miR868-5p**	6.24	**miR319c.2**	0.43	**miR858b**	2.30
**miR1886.2**	1.53	miR394a,b-5p	8.06	**miR866-5p**	8.76
**miR2111b-3p**	4.77	**miR395a,d,e**	6.03	**miR2111a-5p,b-5p**	8.34
**miR3434-3p**	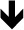	**miR395b,c,f**	3.29	**miR2111b-3p**	33.06
**miR5027**	8.75	miR397a	4.20	**miR2936**	16.25
**miR5634**	0.42	miR399a	5.53	**miR3434-5p**	0.12
**miR5648-3p**	1.66	miR398b-3p,c-3p	2.65	**miR5639-5p**	4.38
**miR5655**	1.82	miR399b,c-3p	3.72	**miR5648-3p**	1.96
**miR8175**	1.89	miR399f	8.08	**miR5654-3p**	1.77
		miR408-5p	2.88		

*MiR3434-3p level decrease could not be determined due to no read counts obtained under stress conditions. New drought stress responsive Arabidopsis miRNAs unknown until now are shown in bold. FC means fold change*.

Northern blot hybridization was carried out for selected miRNAs to confirm their responsiveness under drought stress. Special attention was paid to recently discovered Arabidopsis microRNAs like miR5655 for which the NGS data indicated an increased level under mild drought stress conditions. Northern hybridization confirmed an elevated level of miR5655 in both stress conditions (Figure 2). There was also an increase in abundance observed for the miR2933b using both NGS and northern hybridization (Figure 2). MiR396b-3p was also hybridized under drought conditions since it was one of the miRNA stars affected by heat stress and it was interesting to learn about its expression pattern under drought as another climate change related stress. This miRNA happened to be induced under mild drought stress conditions (while the NGS data was insignificant it shows the same tendency as Northern hybridization results) (Figure 2). Severe drought led to an increased level of sulfate-deprivation-inducible (Jagadeeswaran et al., 2014) Arabidopsis miR395 (NGS data), which was confirmed by Northern hybridization (Figure 2) and TaqMan® miRNA395b assay (Figure S2).

#### Heat-responsive new microRNAs

Heat stress impact on *A. thaliana* seedlings resulted in a similar, as for other stresses, low number of mature miRNAs responding under heat stress duration (13 miRNAs, Table 4, Tables S3C,D). Usually half of the observed expression changes referred to the so called miRNA stars that were mostly downregulated while their cognate microRNAs remained unchanged (Table 4). Mostly their level was decreased in time course of heat stress. Single cases of miRNA and miRNA^*^ upregulation in heat were found (Figure 2, Table 4). The level changes of microRNA^*^s in heat stress are unusual in comparison to other stresses analyzed using the same approach and may point to different processing and stability of microRNA^*^s and/or microRNAs upon high temperature.

**Table 4:** **0.5 h (H 0.5) and 6 h (H 6) heat stress affected** ***Arabidopsis*** **miRNAs revealed by high-throughput small RNA NGS**.

**H 0.5**		**H 6**	
**MiRNA**	**FC**	**MiRNA**	**FC**
**miR156f-3p**	4.90	**miR156g**	0.79
miR156h	3.73	**miR164b-3p**	0.22
**miR157a-3p,b-3p**	2.19	**miR164c-3p**	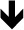
**miR161.2**	0.75	**miR165a-5p**	0.11
**miR165a-5p**	0.60	**miR166a-5p,b-5p**	0.08
**miR166a-5p,b-5p**	0.40	**miR166e-5p**	0.12
**miR166e-5p**	0.44	**miR167a-3p**	0.06
**miR171a-5p,170-5p**	0.26	**miR168a-5p,b-5p**	0.61
**miR171b-5p**	0.45	**miR168a-3p**	0.33
**miR173-5p**	0.78	**miR171b-5p**	0.24
**miR173-3p**	0.64	**miR172e-3p**	0.63
**miR319a.2**	0.55	**miR173-3p**	0.36
**miR319b.2**	0.62	**miR319b.2**	0.55
**miR391-5p**	0.45	**miR391-3p**	0.54
**miR391-3p**	0.40	**miR394a,b-5p**	2.25
**miR396a-5p**	0.57	**miR396a-5p**	0.70
**miR403-3p**	0.78	**miR396a-3p**	0.54
**miR447a-3p,b**	0.80	miR398b-5p,c-5p	0.36
**miR472-5p**	0.40	**miR472-5p**	0.41
**miR472-3p**	0.90	**miR822-3p**	0.48
**miR824-3p**	1.20	**miR824-3p**	2.35
**miR825**	0.68	**miR842**	0.56
**miR841b-3p**	0.61	**miR861-5p**	0.11
**miR1886.1**	0.32	**miR861-3p**	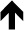
**miR3434-5p**	0.06	**miR862-3p**	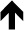
**miR3440b-3p**	0.66	**miR3434-5p**	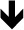
**miR5012**	0.15	**miR3440**	0.71
**miR5014a-5p**	0.07	**miR5020b**	0.41
**miR5024-5p**	1.52	**miR8174**	0.20
**miR5661**	0.76	**#miR393a-5p,b-5p**	1.61
**^#^miR171b-3p,c-3p**	0.54		

*NGS results were suggested by One-Way Anova test (p ≤ 0.05). MiRs: 164c-3p, 861-3p, 862-3p, 3434-5p level changes could not be determined due to no read counts obtained under stress conditions. New heat stress responsive Arabidopsis miRNAs unknown until now are shown in bold. For # depicted miRs NGS results were suggested by One-Way Anova test (p ≤ 0.1). FC means fold change*.

#### Salinity responsive new microRNAs

Numerous salinity stress responsive miRNAs are known for Arabidopsis (Liu et al., 2008). However, the NGS data herein revealed an additional 57 new miRNAs with their expression affected in 2-week old seedlings (Table S3E). Table 5 presents the most profoundly changed salinity-responsive miRNAs using NGS and suggested by One-Way Anova test (*p* ≤ 0.05). Figure 2 shows Northern hybridization confirming increased levels of the selected miRNAs: 157, 162, and miR775 upon salinity stress. Other salinity responsive miRNAs with a less profound fold change but still statistically significant can be found in Table S3E. Almost half of the salinity stress-responsive miRNAs were also found among microRNAs with their expression profile changed in drought. This supports the existence of a cross-talk between metabolic pathways involved in both abiotic stresses.

**Table 5:** **Salinity (NaCl+) stress affected** ***Arabidopsis*** **miRNAs revealed by high-throughput small RNA NGS**.

**NaCl+**					
**MiRNA**	**FC**	**MiRNA**	**FC**	**MiRNA**	**FC**
miR156a-3p	2.18	**miR396a-3p**	3.80	**miR858b**	3.38
miR156b-3p	2.33	miR396a-5p	11.66	**miR861-5p**	0.18
miR156c-3p	1.54	miR396b-5p	3.39	**miR861-3p**	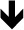
**miR157a-5p,b-5p,c-5p**	1.20	**miR396b-3p**	3.22	**miR2111b-3p**	7.63
miR159a	2.11	miR397a	2.04	**miR2111a-5p,b-5p**	2.52
miR159b-3p	2.89	**miR399a**	3.97	**miR2936**	78.84
**miR160a-3p**	0.21	**miR399c-5p**	6.39	**miR3434-5p**	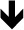
**miR162a-3p,b-3p**	1.34	**miR399d**	3.56	**miR3440b-5p**	18.65
**miR164c-3p**	4.49	miR408-3p	4.43	**miR3932b-5p**	5.58
miR165a-5p	0.39	**miR447a.2-3p**	2.36	**miR4228-3p**	1.87
miR166a-5p,b-5p	0.20	**miR447c-5p**	2.73	**miR5023**	0.32
miR166e-5p	0.33	**miR771**	0.23	**miR5028**	2.93
miR167a-3p	4.70	**miR775**	1.75	**miR5632-3p**	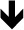
miR169b-5p,c	0.05	**miR780.2**	0.14	**miR5638a**	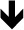
miR169h,i,j,k,l,m,n	0.41	**miR823**	2.70	**miR5638b**	0.18
miR171a-3p	2.03	**miR824-3p**	2.73	**miR5642a,b**	6.65
miR171b-3p,c-3p	2.88	**miR829-3p.1**	0.44	**miR5648-5p**	6.43
**miR172b-5p,e-5p**	4.01	**miR831-5p**	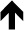	**miR5650**	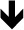
**miR172c,d-3p**	0.46	**miR833b**	2.94	**miR5653**	1.55
**miR172e-3p**	0.42	**miR834**	2.90	**miR5999**	1.61
miR319a,b	4.74	**miR843**	1.57	**miR8176**	70.60
**miR319b.2**	0.45	**miR850**	1.53	**miR8181**	0.21
**miR319c.2**	0.48	**miR851-3p**	0.23		

*NGS results were suggested by One-Way Anova test (p ≤ 0.05). MiR 831-5p level could not be determined due to no read counts obtained under control conditions. MiRs: 861-3p, 3434-5p, 5632-3p, 5638a, 5650 levels could not be determined due to no read counts obtained under stress conditions. New salinity stress responsive Arabidopsis miRNAs unknown until now are shown in bold. FC means fold change*.

#### MicroRNAs responsive to copper deficiency

Thirty six new copper deficiency responsive miRNAs were identified when NGS approach was used (Table S3F, Table 6). The elevated level of a few miRNAs selected from the above group was confirmed by Northern hybridization (Figure 3) and TaqMan® miRNA assays (Figure S2). The increased level of the most important known Cu deficiency responsive miR398 (Sunkar et al., 2012) was also verified by Northern hybridization (data not shown; the tendency of the level change obtained by NGS for this miRNA—its upregulation—was maintained, although its change was not statistically significant). From the metal stresses analyzed, copper deficiency stress led to the largest number of mature miRNA responses suggesting serious disturbances in plant metabolism upon the lack of copper.

**Table 6:** **Copper deficiency (Cu−), copper excess (Cu+), cadmium excess (Cd+), and sulfur deficiency (S−) stresses affected** ***Arabidopsis*** **miRNAs revealed by high-throughput small RNA NGS**.

**Cu−**		**Cu+**		**Cd+**	
**MiRNA**	**FC**	**MiRNA**	**FC**	**MiRNA**	**FC**
**miR159a**	1.84	**miR169d,e,f-5p,g-5p**	0.79	**miR156f-3p**	2.84
**miR159b-3p**	2.44	**miR169a-3p**	0.29	**miR157a-3p,b-3p**	2.22
**miR162a,b-5p**	1.27	**miR319a,b**	3.27	**miR159b-3p**	2.11
**miR165a-3p,b**	1.56	**miR394a,b-5p**	3.59	**miR167c-3p**	1.58
**miR166a-3p,b-3p,c,d,e-3p,f,g**	1.51	**miR400**	0.78	**miR319a,b**	3.14
**miR167c-3p**	1.86	**miR771**	0.48	**miR396b-3p**	1.90
**miR168b-3p**	2.29	**miR829-5p**	0.25	**miR400**	0.47
**miR169g-5p,f-5p,d,g**	0.70	**miR837-3p**	0.85	**miR847**	2.52
**miR171a-5p**	3.40	**miR841a-3p**	1.56	**miR851-3p**	2.19
**miR319a,b**	2.36	**miR843**	1.93	**miR3440b-5p**	5.29
**miR319b.2**	1.40	**miR847**	2.25	**miR5640**	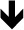
**miR396a-3p**	1.99	**miR5632-3p**	1.90	**miR5641**	0.49
**miR400**	0.54	**miR5664**	1.58	**miR5996**	1.21
**miR472-3p**	1.98	**miR8175**	0.49	**miR8175**	0.43
**miR781a,b**	0.34	**S−**			
**miR830-5p**	0.16	**MiRNA**	**FC**	**MiRNA**	**FC**
**miR843**	1.60	**miR173-5p**	0.77	**miR2111a-3p**	2.04
**miR847**	3.14	miR395a,d,e	2.13	**miR5632-3p**	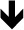
**miR5012**	0.21	**miR319b.2**	1.22	**miR5638b**	0.44
**miR5642a,b**	1.63	**miR403-5p**	2.09	**miR8172**	0.45
**miR8171**	1.42	**miR771**	0.28		
**miR8175**	0.29	**miR2111a-5p,b-5p**	1.27		

*NGS results were suggested by One-Way Anova test (p ≤ 0.05). MiRs: 5632-3p and 5640 levels could not be determined due to no read counts obtained under stress conditions. New Cu−, Cu+, Cd+, S− stresses responsive Arabidopsis miRNAs unknown until now are shown in bold. FC means fold change*.

#### MicroRNAs responsive to copper excess

All identified miRNAs responding to Cu excess by NGS approach (*p* ≤ 0.05) are presented in Table S3G, while Table 6 shows examples of new miRNAs most profoundly responsive to Cu toxicity detected in Arabidopsis 15-day old seedlings. Several miRNAs that responded significantly to Cu deficiency stress were also responsive under Cu excess (miR165ab, miR166, miR319, miR400). This suggests their general role in the copper homeostasis maintenance in Arabidopsis. The level changes of these miRNAs were confirmed by Northern hybridization (Figure 3). Twenty-four hours Cu excess stress treatment of Arabidopsis seedlings had less of an impact on changes in miRNA levels than it was in the case of Cu deprivation stress. This also suggests the more deleterious effect of copper deficiency than copper excess on plant growth and development (Abdel-Ghany and Pilon, 2008).

#### MicroRNAs responsive to cadmium excess

Over 50 new cadmium responsive miRNAs were detected by NGS approach (Table S3H). Examples of the newly discovered miRNAs that responded the most profoundly to Cd toxicity are shown in Table 6. The level changes of selected miRNAs were also supported by Northern hybridization (Figure 3). Additionally, miR164 was tested, since it was previously reported as a potential novel Cd responsive miRNA in soybean (NGS data show the same tendency but are statistically insignificant) (Fang et al., 2013). MiR173 that is known to be involved in *trans*-acting small interfering RNAs (ta-siRNAs) biogenesis (Yoshikawa et al., 2013) was also tested, since ta-siRNAs production involved miRNA390 was reported to be repressed in roots upon Cd toxicity (Mendoza-Soto et al., 2012). MiR164 and miR173 were affected (increased and decreased, respectively) upon Cd stress in Arabidopsis seedlings while the level of miR390 was not changed (data not shown). The results show that cadmium excess stress exerts a broad miRNA response in Arabidopsis. This suggests that although cadmium is a deleterious toxic agent in general, it can possibly induce several serious miRNA-guided metabolic rearrangements, helping a plant to survive in the cadmium polluted soil.

#### MicroRNAs responsive to sulfur deficiency

It is well established that miR395 is induced during sulfur deprivation in Arabidopsis (Jagadeeswaran et al., 2014). The small RNA NGS results obtained in this study for sulfur deficiency treated Arabidopsis 14 day-old seedlings confirmed miR395 induction. Generally, the NGS revealed a low number of affected miRNAs and additionally subtle level changes to sulfur deprivation (0.5 ≤ fold change ≤ 1.5) (Table S3I). Only a few miRNAs: 395a,d,e, 403-5p, and 2111a-3p had a two-fold higher level in comparison to control conditions (Table 6). MicroRNAs that we have shown to be responsive to other abiotic stresses were tested for their response to sulfur deficiency. The Northern hybridization showed miR162 induction and miR173 downregulation (Figure 3, NGS data show the same tendency but are statistically insignificant). These studies show that Arabidopsis miRNAs are not generally sensitive to sulfur deficiency.

### General stress-responsive microRNAs

**Figure 4:**
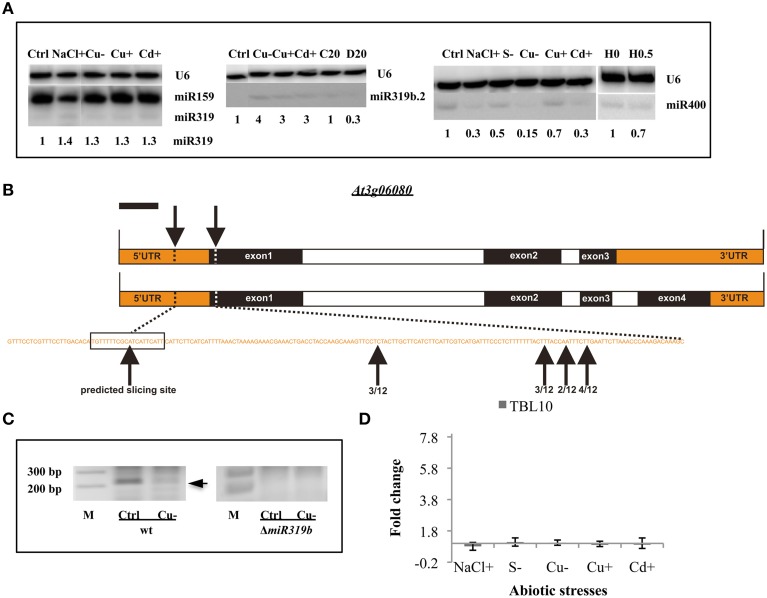
**General stress-responsive miRNAs**. **(A)** General abiotic stress responsive *Arabidopsis thaliana* miRNAs 319a/b, 319b.2, and 400 revealed by Northern hybridization under different abiotic stresses. The white lines separate signals from miRNAs and U6snRNA loading control as well as both signals for the following stress samples: NaCl+ and Cu− (for miR319a/b), control (Ctrl) and Cu− (for miR319b.2), Cd+ and H 0 (for miR400), all probed on the same blots. **(B)** The structure of the *TBL10* and the predicted as well as 5′RACE identified slicing sites within its mRNAs. **(C)** The agarose gels showing the miR319b.2 directed 3′-TBL10 mRNA cleavage products in wt plants and Δ*miR319b* mutant plants. Arrow points to the expected length of the 5′RACE product. **(D)** The RT-qPCR of the *TBL10* expression in wt plants under different abiotic stress conditions. Values on the chart are shown as the mean ± SD relative expression level from three independent experiments. Symbols representing various stresses are marked as described in the Figure 1.

Throughout the experiments, miR319a/b, miR319b.2, and miR400 manifested as multi-stress responsive miRNAs (Figures 3, 4A). MiR400 happened to be decreased in all stress experiments conducted on Arabidopsis seedlings (Figures 3, 4A, Table 7). MiR319b.2 was increased in response to copper, cadmium, and sulfur deficiency stresses (Figures 3, 4A). However, miR319b.2 levels decreased in response to severe drought conditions, heat, and salinity stresses (Table 7, Figure 4A). The levels of miRNA319a/b, like miR319b.2, were elevated under metal stresses. In contrast to miR319b.2, the levels of miRNA319a/b were also highly increased under salinity stress conditions.

**Table 7:** **General stress-responsive** ***Arabidopsis*** **microRNAs revealed by high-throughput small RNA NGS**.

**Stress**	**MiRNA**	**Fold change (NGS)**	***p*-value**
NaCl+	**miR400**	0.86	0.18
S**−**		0.79	0.14
Cu−		0.54	**0.003**
Cu+		0.78	**0.05**
Cd+		0.47	**0.002**
0.5 h heat		0.63	0.18
NaCl+	**miR319b.2**	0.45	**0.003**
S**−**		1.22	**0.03**
Cu−		1.40	**0.01**
Cu+		1.05	0.68
Cd+		1.19	0.27
20%SWCdrought		0.60	**0.01**
0.5 h heat		0.62	**0.02**
6 h heat		0.55	**0.001**
NaCl+	**miR319a,b**	4.74	**1.5E-05**
Cu−		2.36	**0.02**
Cu+		3.27	**0.02**
Cd+		3.14	**0.005**

*One-Way Anova test (p ≤ 0.05) statistically significant results are shown in bold*.

The previously identified putative miRNA319b.2 target gene (Sobkowiak et al., 2012) *At3g06080* mRNA that codes for Trichome Birefringence-like 10, TBL10 protein, was confirmed experimentally under copper deficiency stress. TBL10 belongs to the TBL protein family composed of 46 members that contain a plant-specific DUF231 domain. It has been shown that some members of this family are required for cellulose biosynthesis in Arabidopsis (Bischoff et al., 2010). Figure 4B shows the structure of the *TBL10* gene, which actually encodes two transcripts differing in the presence or lack of exon 4. Both mRNAs can be targeted by miR319b.2 within the 5′-UTR region (Figure 4B) (Sobkowiak et al., 2012). Using 5′RACE approach, it was aimed to experimentally confirm that TBL10 mRNAs could be cleaved by miR319b.2-guided RISC complex at the predicted site. The 5′RACE results showed mRNA fragments cut close but always downstream from the putative slicing site but never upstream of the predicted slicing site (Figure 4B). It was decided to compare the amount of putative TBL10 mRNA cleavage products in wt and Δ*miR319b* mutant plants under control and Cu deficiency conditions. The cleavage product was present under control conditions and to a lesser extent under Cu deficiency stress only in wt plants (Figure 4C). This experiment proves that the obtained 5′RACE products are linked to the presence of the miR319b.2. The level of the TBL10 mRNA in wt plants under different abiotic stresses studied was tested. RT-qPCR was performed using primers flanking the predicted slicing site for miRNA319b.2. Although the expected result was the downregulation of TBL10 target under Cu deficiency stress, the constant expression of *TBL10* was encountered there. The same results were obtained for other stresses applied to Arabidopsis 14-day old seedlings, where the level of miR319b.2 was increased. TBL10 mRNA level remained unchanged under different abiotic stresses can be the result of the fine tunning its expression. Presumably in the case of Cu deficiency, it is due to the presence of 3 CuREs (Copper Responsive Elements, GTAC sequences) within a 2.5 kb sequence upstream from TBL10 5′UTR, that contain important features in Cu responsiveness (Higo et al., 1999; Yamasaki et al., 2009).

Identification of general abiotic stress responsive microRNAs implies their involvement in the regulation of metabolic pathways that are activated upon different environmental stimuli.

## Discussion

Studies presented here concerned individually applied stresses and their impact on miRNA profiles in Arabidopsis. Stress-induced changes in microRNA levels will affect target gene expression. Target genes are mainly transcription factors thus a cascade of genes will be regulated influencing the basic functions of a plant.

The pri-miRNA expression changes observed under the different abiotic stresses here studied did not allow the prediction of the level of specific mature miRNAs. The detected lack of correlation between the pri-miRNAs and the abundance of mature miRNAs also varied significantly between stresses applied (Table 2, Figure S2). This might be due to extensive posttranscriptional regulation of microRNA biogenesis. Many microRNA genes are long and contain multiple introns. Therefore, the pri-miRNAs processing might be regulated by splicing, alternative splicing, alternative polyA site selection, pre-miRNA susceptibility to be processed to the mature microRNA and finally microRNA stability (Kai and Pasquinelli, 2010; Bielewicz et al., 2012; Yan et al., 2012; Köster et al., 2014). The proper 5′ splice site selection of a downstream intron and proximal, intronic polyA site selection in miR163 precursor determines pathogen-triggered accumulation of miR163 in Arabidopsis (Bielewicz et al., 2013; Szweykowska-Kulińska et al., 2013). Pri-miRNA structures also play regulatory role in plant response to hormone signaling. This is demonstrated by the generation of miR846 and miR842 from alternatively spliced isoforms. Both are functionally related, and their levels are regulated by abscisic acid (ABA). Upon exogenous ABA application to Arabidopsis seedlings, the level of miR846 is decreased. This level reduction is accompanied by a decrease in the level of an alternatively spliced isoform from which miR846 can be generated (Jia and Rock, 2013). The regulation of miRNA levels by alternative splicing can be induced by heat stress. MiR400 is encoded within an intron of a protein-coding gene and its level is decreased upon heat stress. High temperature induces alternative splicing that shifts miR400 from intronic to exonic localization, which inhibits its efficient processing from the stress-induced mRNA isoform (Yan et al., 2012). In our studies miR400 level is downregulated in various stresses including heat while its pri-miRNA is upregulated or remains constant, suggesting an important role of posttranscriptional regulation in this miRNA biogenesis.

Numerous new Arabidopsis miRNAs were detected in the present study. Some of them were already reported as stress-responsive miRNAs in other plant species, however many of newly discovered Arabidopsis miRNAs were never addressed concerning environmental stresses. It will be interesting to learn about the role of proteins encoded by mRNAs targeted by these novel, stress-responsive miRNAs in plant response to changing milieu.

Abiotic stresses generally induce the accumulation of reactive oxygen species (ROS) (Mittler, 2002), although it depends on the type and intensity of the stressor (Kruszka et al., 2012). Ozone is a model abiotic elicitor of ROS and 22 Arabidopsis ozone responsive miRNAs were identified, out of which 20 overlapped with cold, salt, osmotic and UV-B radiation stress responsive miRNAs (Iyer et al., 2012). Arabidopsis root hypoxia-responsive microRNAs were also identified and linked to developmental responses to low oxygen dependent on mitochondrial function (Moldovan et al., 2010), although another report stated that regulation of gene expression via miRNAs appeared to play a minor role during hypoxia (Licausi et al., 2011). It is well known that ROS plays a central role in coordinating defense signaling in plants in response to both biotic and abiotic stresses (Iyer et al., 2012). In the studies presented here the ROS responsive miRNAs (Iyer et al., 2012) could be identified as overlapping with those responding to different abiotic stresses analyzed (Tables 3–7). They included the members of such miRNA families, like miR156, 157, 159, 160, 164, 165, 166, 167, 168, 169, 171, 319, 394, 398 and 403 and supported the cross talk between different stress signaling pathways, probably mediated by ROS.

We also revealed four examples of miRNAs responding to a wide array of abiotic stresses: miR319a/b, miR319b.2, and miR400. Their broad response may be due to the numerous promoter response elements (RE) that can be found upstream of these *MIR* genes. For example *MIR*319b promoter contains 3 CCAAT boxes (heat shock RE), 5 MYC and at least 6 MYB elements (dehydration RE), at least 7 NGATT (RE controlling responses to a variety of environmental stimuli), 5 ACGT—ERD1 RE (early response to dehydration), 5 TGACY RE (activation of the ERF3 gene by wounding), and 8 CuREs (copper RE). There are also 1 ERE (ethylene-RE) and 2 GAREs (gibberellins-RE), often present in addition to other cis-acting elements in the promoters of rice metal responsive miRNA genes (Higo et al., 1999; Ding et al., 2011). However, transcriptional regulation is often followed by posttranscriptional control. For example the levels of miR319a/b are upregulated in salinity stress while the level of the miR319b.2 that is derived from the same precursor as the miR139b, is downregulated.

The miR319a/b targets belong e.g., to the family of TCP transcription factors implicated in growth control and therefore point to a role of miR319a/b in leaf development, floral organ identity, and flowering time (Palatnik et al., 2003; Schwab et al., 2005). Since a primary common effect of high concentrations of metals such as aluminum, copper, cadmium, or mercury is on root growth inhibition (Mendoza-Soto et al., 2012), one may presume this might be also due to miRNA319a/b induction under copper or cadmium excess stresses in seedlings. The involvement of miR319b in Arabidopsis copper homeostasis can be also deduced from its promoter sequence containing 8 CuREs (Higo et al., 1999).

Using 5′RACE approach TBL10 mRNA was confirmed as a new target for miR319b.2. However, under different abiotic stresses it did not change its expression level in comparison to control conditions. In the promoter region of the TBL10 gene the presence of many stress responsive elements was recorded, including 3 CuREs, 3 CCAAT sequences, 6 MYB and 8 MYC elements in addition to 6 ERD1 related elements (Higo et al., 1999). They can be found in different metal responsive genes including those encoding metal responsive miRNAs: 319, 390, 393, 398 (Mendoza-Soto et al., 2012). Therefore, we suggest that under abiotic stresses transcriptional induction of *TBL10* results in an increase of transcript level that is downregulated by an increase of miR319b.2 thereby resulting in a stable level of the TBL10 mRNA. It is known from previous studies that Arabidopsis miR319b.2 targets an intron-containing RAP2.12 mRNA isoform in adult plants (Sobkowiak et al., 2012). TBL10 is one of five protein-coding genes selected as putative targets for miR319b.2 although TBL10 mRNA was not cleaved by the miR319b.2 in the adult plants (Sobkowiak et al., 2012). On the contrary, the TBL10 miR319b.2 target was cleaved in Arabidopsis seedlings while the RAP2.12 mRNA isoform was not (data not shown). One may speculate that miR319b.2 is therefore able to recognize different targets at different Arabidopsis developmental stages and conditions.

Experiments presented in this work add new miRNA players in a complex network of gene expression regulation in plant response to a wide array of abiotic stresses. They include the group of newly identified miRNAs that are specific to particular stress conditions as well as of general multi-stress responsive miRNAs. The strong involvement of the latter to metal stresses may contribute to a better understanding of the roles of plant miRNAs during common stresses imposed on plants.

### Conflict of interest statement

The authors declare that the research was conducted in the absence of any commercial or financial relationships that could be construed as a potential conflict of interest.
