# Supplementary figures and images for: Arabidopsis microRNA expression regulation in a wide range of abiotic stress responses
Source: Front Plant Sci. 2015 Jun 4;6:410. doi: 10.3389/fpls.2015.00410 (PMC4454879; doi:10.3389/fpls.2015.00410)

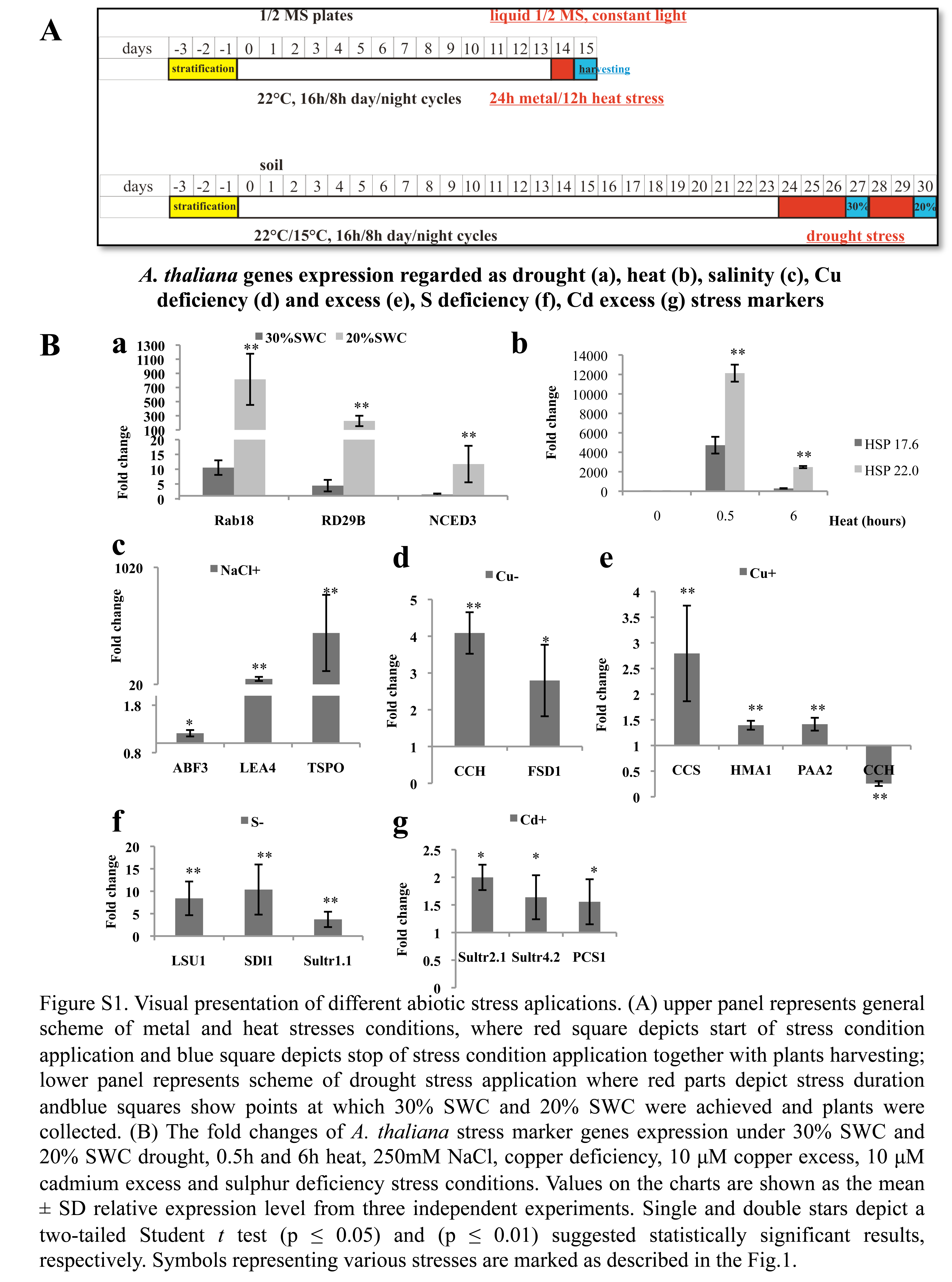

Supplement: Supplementary file 4 [file Image1.TIF]

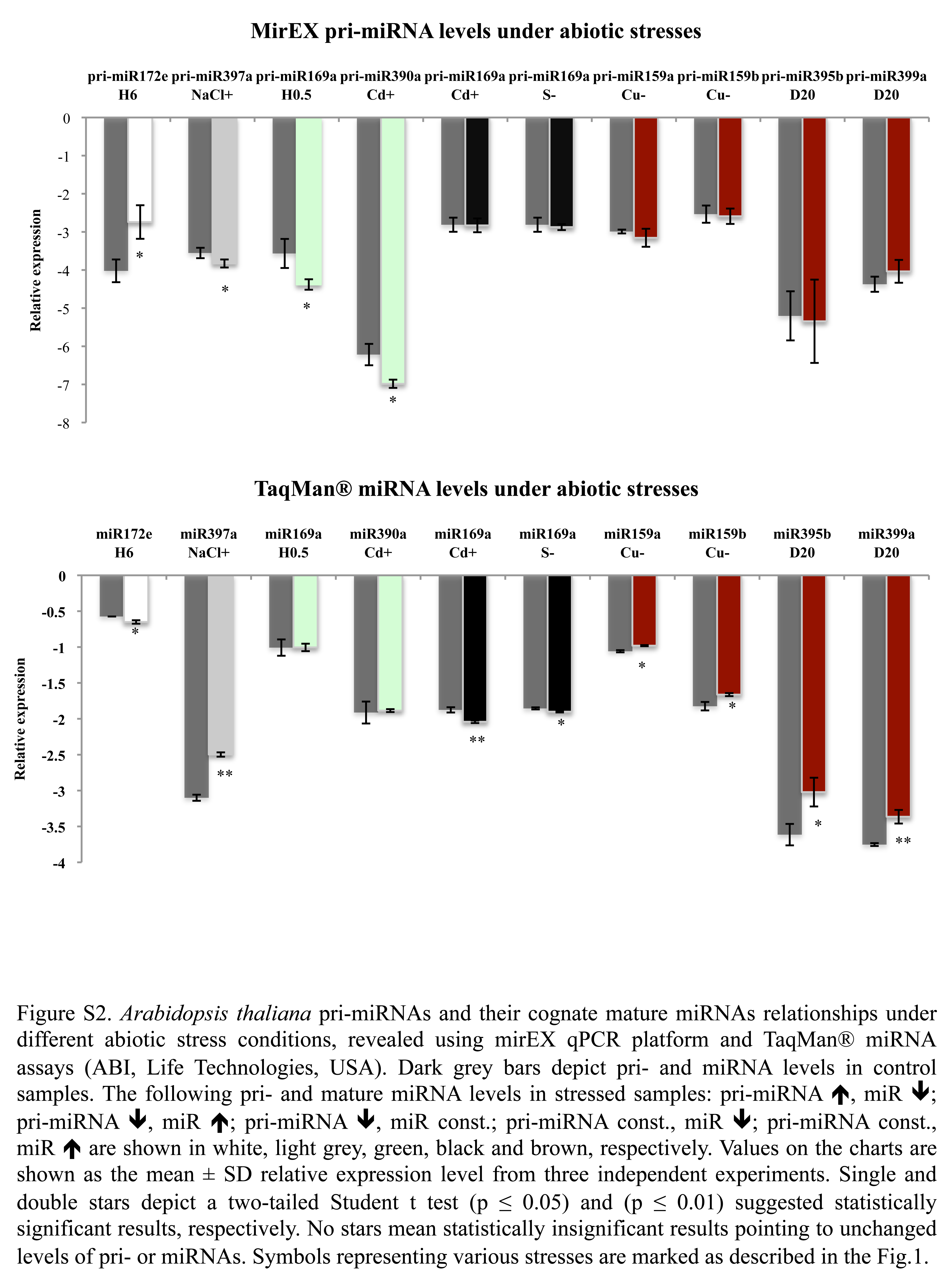

Supplement: Supplementary file 5 [file Image2.TIF]
